# Supplementary material for: Identification of photoperiod-induced specific miRNAs in the adrenal glands of Sunite sheep (Ovis aries)
Source: Front Vet Sci. 2022 Jul 22;9:888207. doi: 10.3389/fvets.2022.888207 (PMC9354845; doi:10.3389/fvets.2022.888207)
Supplement: Supplementary file 1 [file Data_Sheet_1.ZIP › Supplementary file/miRDeep2 file.zip!/miRDeep2 file/Know_Structure/SPLP42A2/oar-mir-369.pdf]

miRBase precursor : oar-mir-369
Total read count : 14012
oar-miR-369-5p read count : 1558
oar-miR-369-3p read count : 12453
remaining reads : 1

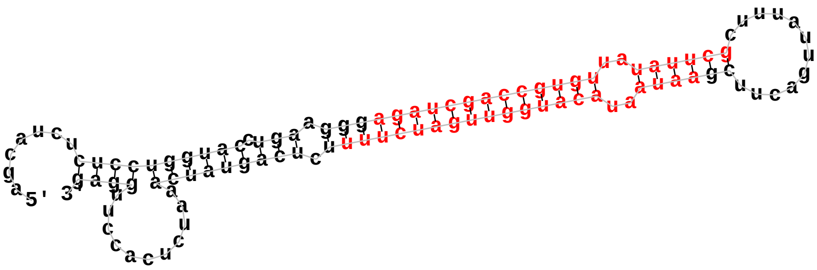

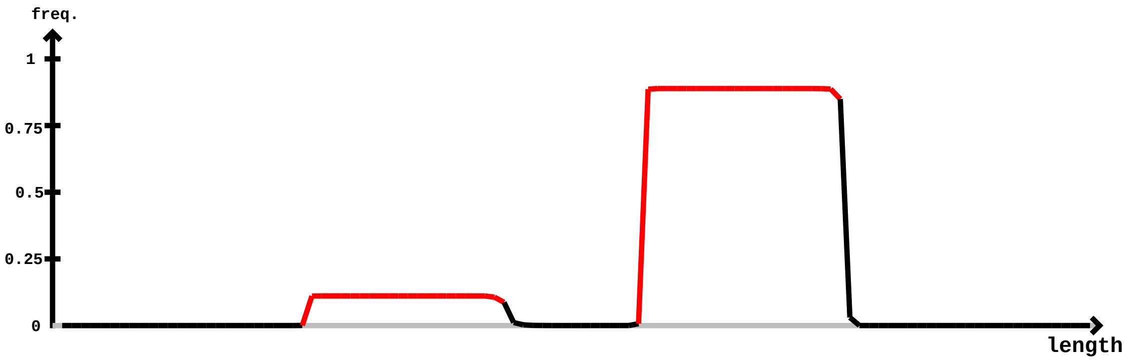


```
                      oar-miR-369-5p
                                                  oar-miR-369-3p
5'- agcaucucuccugguaccugaagggagaucgaccguguuauauucgcuuuauugacuucgaauaauacaugguugaucuuuucucaguaucaaaucucaccuuggag  -3'   exp
    .......((((((((((.(((.((((((((((((((((..((((((............))))))..)))))))))))))))).))))))))).............))))   reads   mm   sample
    ........................gagaucgaccguguuauauu...............................................................   1       0    seq
    ........................gagaucgaccguguuauauucg.............................................................   5       0    seq
    .........................agaucgaccguguuaua.................................................................   1       0    seq
    .........................agaucgaccguguuauau................................................................   1       0    seq
    .........................agaucgaccguguuauauu...............................................................   61      0    seq
    .........................agaucgaccguguuauauuc..............................................................   273     0    seq
    .........................agaucgaccguguuauauucg.............................................................   1051    0    seq
    .........................agaucgaccguguuauauucgc............................................................   119     0    seq
    .........................agaucgaccguguuauauucgcu...........................................................   31      0    seq
    .........................agaucgaccguguuauauucgcuu..........................................................   6       0    seq
    .........................agaucgaccguguuauauucgcuuu.........................................................   4       0    seq
    ..........................gaucgaccguguuauauuc..............................................................   2       0    seq
    ..........................gaucgaccguguuauauucg.............................................................   3       0    seq
    ......................................................acuucgaauaauacaugguugaucuu...........................   1       0    seq
    ..........................................................cgaauaauacaugguugaucuu...........................   1       0    seq
    ...........................................................gaauaauacaugguugaucuu...........................   12      0    seq
    ...........................................................gaauaauacaugguugaucuuu..........................   84      0    seq
    ...........................................................gaauaauacaugguugaucuuuu.........................   1       0    seq
    ............................................................aauaauacaugguugauc.............................   6       0    seq
    ............................................................aauaauacaugguugaucu............................   19      0    seq
    ............................................................aauaauacaugguugaucuu...........................   511     0    seq
    ............................................................aauaauaAaugguugaucuuu..........................   2       1    seq
    ............................................................aauaauacaugguugaucuuu..........................   11376   0    seq
    ............................................................aauaauacaugguugaucuuuu.........................   409     0    seq
    .............................................................auaauacaugguugaucuuu..........................   16      0    seq
    .............................................................auaauacaugguugaucuuuu.........................   13      0    seq
    .............................................................auaauacaugguugaucuuuuc........................   2       0    seq
    ..............................................................uaauacaugguugaucuuu..........................   1       0    seq
```
